# Seroma following transabdominal preperitoneal patch plasty (TAPP): incidence, risk factors, and preventive measures

**DOI:** 10.1007/s00464-017-5912-3

**Published:** 2017-10-26

**Authors:** F. Köckerling, R. Bittner, D. Adolf, R. Fortelny, H. Niebuhr, F. Mayer, C. Schug-Pass

**Affiliations:** 1 Department of Surgery and Center for Minimally Invasive Surgery, Academic Teaching Hospital of Charité Medical School, Vivantes Hospital, Neue Bergstrasse 6, 13585 Berlin, Germany; 2 Hernia Center, Winghofer Medicum, Winghofer Strasse 42, 72108 Rottenburg am Neckar, Germany; 3 StatConsult GmbH, Halberstädter Strasse 40 a, 39112 Magdeburg, Germany; 4 Department of General, Visceral and Oncologic Surgery, Wilhelminenhospital, Montleartstrasse 37, 1160 Vienna, Austria; 5 Hanse-Hernia Center, Alte Holstenstrasse 16, 21031 Hamburg, Germany; 6 Department of Surgery, Paracelsus Medical University, Müllnerhauptstrasse 48, 5020 Salzburg, Austria

**Keywords:** Inguinal hernia, TAPP, Seroma, Complications, Mesh fixation

## Abstract

**Background:**

The reported range of seroma formation in the literature after TEP repair is between 0.5 and 12.2% and for TAPP between 3.0 and 8.0%. Significant clinical factors associated with seroma formation include old age, a large hernia defect, an extension of the hernia sac into the scrotum, as well as the presence of a residual indirect sac. Seroma formation is a frequent complication of laparoendoscopic mesh repair of moderate to large-size direct (medial) inguinal hernia defects. This present analysis of data from the Herniamed Hernia Registry now explores the influencing factors for seroma formation in male patients after TAPP repair of primary unilateral inguinal hernia.

**Methods:**

In total, 20,004 male patients with TAPP repair of primary unilateral inguinal hernia were included in uni- and multivariable analysis.

**Results:**

Univariable analysis revealed the highly significant impact of the fixation technique on the seroma rate (non-fixation 0.7% vs. tacks 2.1% vs. glue 3.9%; *p* < 0.001). Multivariable analysis showed that glue compared to tacks (OR 2.077 [1.650; 2.613]; *p* < 0.001) and non-fixation (OR 5.448 [4.056; 7.317]; *p* < 0.001) led to an increased seroma rate. A large hernia defect (III vs. I: OR 2.868 [1.815; 4.531]; *p* < 0.001; II vs. I: OR 2.157 [1.410; 3.300]; *p* < 0.001) presented a significantly higher risk of seroma formation. Likewise, medial compared to lateral inguinal hernias had a higher seroma rate (OR 1.272 [1.020; 1.585]; *p* = 0.032).

**Conclusions:**

Mesh fixation with tacks or glue, a larger hernia defect, and medial defect localization present a higher risk for seroma development in TAPP inguinal hernia repair.

According to the Guidelines of the International Endohernia Society (IEHS) and the European Association of Endoscopic Surgery (EAES) [1–3], seroma formation is a frequent occurrence after laparoendoscopic groin hernia repair but lacks clinical relevance or significance in most cases. It is advised to explain the possibility of seroma formation to the patient before surgery to prevent anxiety [3]. The main problem with postoperative seroma is the perception by patients and their general practitioners that they represent a persistence or recurrence of the hernia [4]. There are reports of seroma being mistaken for recurrences following laparoscopic hernia repair, with the correct diagnosis being made only after groin exploration [4].

The reported range of seroma formation in literature reviews after TEP repair is between 0.5 and 12.2% [5] and for TAPP between 3.0 and 8.0% [4]. In a meta-analysis of randomized controlled trials, a significantly higher incidence of seromas was found after laparoendoscopic inguinal hernia repair, with 1590 patients, versus Lichtenstein repair, with 1620 patients (12.2 vs. 8.9%; *p* = 0.003) [6]. If no measures are taken for prevention of seroma after TEP or TAPP repair for direct inguinal hernia, the incidence reported is 4–5% [7]. In large registry studies of primary unilateral inguinal hernia repair in men with the laparoendoscopic technique, the incidence of seroma has been reported as 0.5% for TEP and 3% for TAPP [8, 9]. Significant clinical factors associated with seroma formation include old age, a large hernia defect, an extension of the hernia sac into the scrotum, and the presence of a residual distal indirect sac [10, 11]. Seroma formation is a frequent complication of laparoendoscopic mesh repair of moderate to large size direct medial inguinal hernia defects [5].

While certain studies had identified significantly less seroma formation on using extra lightweight meshes [12], a meta-analysis did not note any difference in the impact of lightweight vs heavyweight meshes on the seroma rate after laparoendoscopic inguinal hernia repair [13]. Likewise, while in one observational study comparison of mesh fixation versus non-fixation for endoscopic inguinal hernia repair revealed a lower seroma rate for non-fixation [14], this was not demonstrated by a corresponding meta-analysis [15]. Besides, comparison of mesh fixation with tacks versus glue did not show any significant difference in the seroma rate [16–18]. In two observational studies, preperitoneal drainage was found to have a positive effect on the seroma incidence [19, 20].

This present analysis of data from the Herniamed Hernia Registry now explores the influencing factors for seroma formation after TAPP repair. Preventative measures will then be discussed.

## Materials and methods

**Fig. 1:**
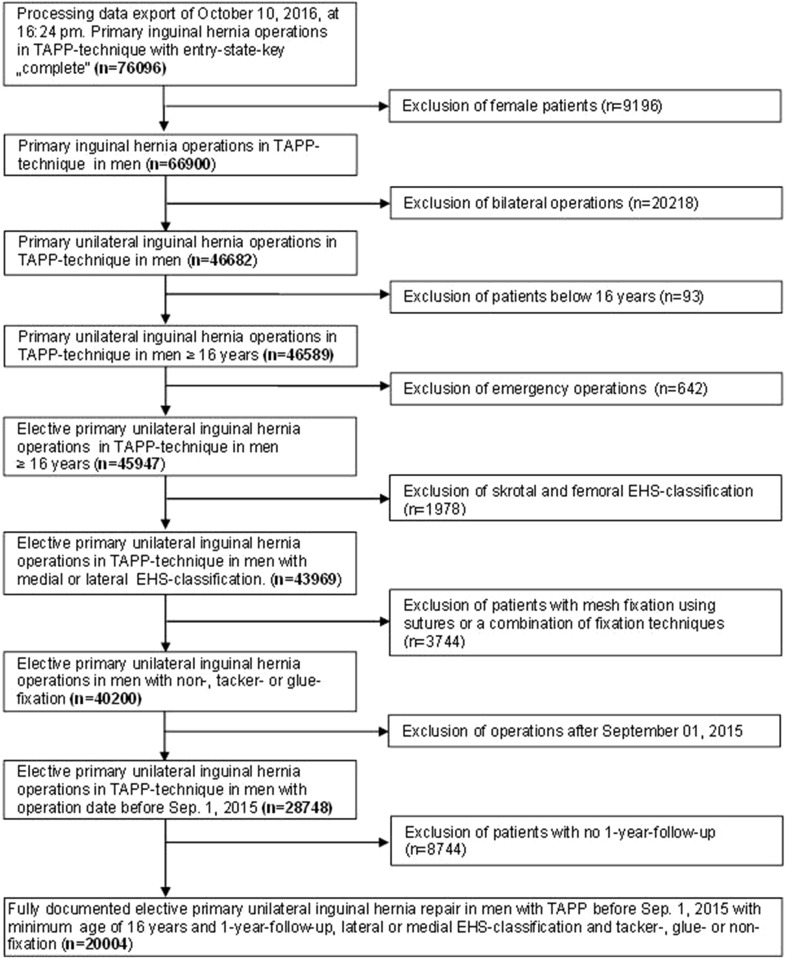
Flowchart of patient inclusion

As of October 10, 2016, 577 participating hospitals and office-based surgeons mainly from Germany, Austria, and Switzerland have entered prospective data into the multicenter internet-based Herniamed Hernia Registry on their patients who had undergone routine hernia surgery and signed an informed consent agreeing to participate [21]. As part of the information provided to patients regarding participation in the Herniamed Quality Assurance Study and signing the informed consent declaration, all patients are informed that the treating hospital or medical practice would like to be informed about any problems occurring after the operation and that the patient has the opportunity to attend clinical examination. All postoperative complications occurring up to 30 days after surgery are recorded. This present study analyzed the prospective data collected for all male patients who had been operated on with a TAPP technique for repair of a primary unilateral inguinal hernia in the period September 1, 2009, up to and including September 1, 2015. At 1-year follow-up, the general practitioners and patients were asked once again for any postoperative complication. If complications are reported by the general practitioner or patient, patients can be requested to attend clinical and/or radiological examinations. A recent publication has provided impressive evidence of the role of patient-reported outcomes [22]. Only those patients for whom 1-year follow-up results were available were included in the analysis. Other inclusion criteria were as follows: age ≥ 16 years and only medial/lateral/combined types of inguinal hernia based on the EHS classification [23]. In total, 20,004 patients were included in uni- and multivariable analysis (Fig. 1) for investigation of influencing factors for the development of a seroma after TAPP inguinal hernia repair. During the observation period, the 20,004 TAPP procedures were performed in 8799 patients (44.0%) without mesh fixation, in 6387 patients (31.9%) using tacks for fixation, and in 4818 patients with glue fixation (24.1%).

The most commonly used meshes (≥ 2%) are given in Table 1. These were mainly large-pore lightweight meshes. The most commonly used (≥ 2%) tacks and glues are listed in Tables 2 and 3, respectively.

**Table 1:** Proportion of meshes most commonly used (≥ 2%)

Type of mesh	Type of fixation						Total	
	Non-fixation		Tacks		Glues			
	*N*	%	*N*	%	*N*	%	*N*	%
Prolene	12	0.1	447	7.0	6	0.1	465	2.3
Ultrapro	1139	12.9	1770	27.7	2112	43.8	5021	25.1
Parietene standard	297	3.4	240	3.8	10	0.2	547	2.7
Parietex ProGrip	1017	11.6	11	0.2	8	0.2	1036	5.2
Parietene ProGrip	1332	15.1	29	0.5	1	0.0	1362	6.8
Parietene light	468	5.3	414	6.5	70	1.5	952	4.8
DynaMesh-ENDOLAP	219	2.5	366	5.7	409	8.5	994	5.0
TiMesh extralight	260	3.0	85	1.3	383	7.9	728	3.6
TiMesh light	646	7.3	569	8.9	222	4.6	1437	7.2
3DMax light	1007	11.4	398	6.2	146	3.0	1551	7.8
Optilene	129	1.5	226	3.5	208	4.3	563	2.8
Optilene LP	326	3.7	651	10.2	733	15.2	1710	8.5
TiO2Mesh	460	5.2	86	1.3	115	2.4	661	3.3
Other meshes	1487	16.9	1095	17.1	395	8.2	2977	14.9
Total	8799	100.0	6387	100.0	4818	100.0	20,004	100.0

**Table 2:** Proportion of tacks most commonly used (≥ 2%)

Type of tacks	*N*	%
ProTack	348	5.4
Endo universal	531	8.3
AbsorbaTack	2845	44.5
PermaSorb	318	5.0
EMS stapler	1299	20.3
SorbaFix	568	8.9
SecureStrap	328	5.1
Others	150	2.3
Total	6387	100.0

**Table 3:** Proportion of glue most commonly used (≥ 2%)

Type of glue	*N*	%
Tissucol/tisseel	2050	42.5
Glubran 2	652	13.5
Evicel	1607	33.4
Liquiband FIX8	161	3.3
Others	348	7.2
Total	4818	100.0

All analyses were performed with the software SAS 9.4 (SAS institute Inc. Cary, NC, USA) and intentionally calculated to a full significance level of 5%, i.e., they were not corrected in respect of multiple tests, and each *p* value 0.05 represents a significant result. To first discern the differences between the groups in unadjusted analyses. Fisher’s exact test was used for categorical outcome variables, and the robust *t* test (Satterthwaite) for continuous variables. For mesh size (cm^2^), a logarithmic transformation was applied and re-transformed mean and range of dispersion are given.

To identify influence factors in multivariable analysis of seroma, a binary logistic regression model was used. Potential influence factors were: ASA score (I/II/III/IV), age (years), BMI (kg/m^2^), mesh size (cm^2^), defect size (I/II/III), risk factors (yes/no), preoperative pain (yes/no/unknown), EHS classification (lateral/medial/combined), drainage (yes/no), and mesh fixation (non-fixation/tacks/glue). Estimates for odds ratio (OR) and the corresponding 95% confidence interval based on the Wald test were given. For influence variables with more than two categories, pairwise odds ratios were given. For age (years), the 10-year OR estimate, for BMI (kg/m^2^) the five-point OR estimate, and for mesh size (cm^2^) the 10-point OR estimate were given. Results are presented in tabular form, sorted by descending impact.

## Results

### Univariable analysis

Univariable analysis of the relationship between the fixation technique (non-fixation/tacks/glue) and the patient and operative characteristics revealed highly significant differences (Tables 4, 5). For example, the proportion of patients with tacks or glue fixation for large hernia defects (EHS III > 3 cm) was significantly greater than that with non-fixation (Table 5). Likewise, the proportion of medial EHS classifications was higher in the tacks and glue mesh fixation group than in the no mesh fixation group. Drain placement was more common in cases of no mesh fixation (Table 5). With regard to mesh fixation (non-fixation/tacks/glue), highly significant differences were seen in the overall postoperative complication rate (non-fixation 1.8% vs. tacks 3.0% vs. glue 4.8%; *p* < 0.001) (Table 6). Seroma accounted for the greatest proportion of postoperative complications (non-fixation 0.7% vs. tacks 2.1% vs. glue 3.9%); *p* < 0.001).

**Table 4:** Mean age, BMI, and mesh size in male patients with primary unilateral inguinal hernia repair in TAPP technique

	Non-fixation	Tacker	Glue	*p*
Age (years)				
Median ± STD	55.0 ± 15.6	58.8 ± 14.7	56.4 ± 15.0	< .001
BMI				
Mean ± STD	25.9 ± 3.3	26.0 ± 3.4	25.8 ± 3.4	< .001
Mesh size (cm^2^)				
Mean (range of dispersion)	146.3 (145.2; 147.5)	149.9 (148.7; 151.1)	151.1 (150.1; 152.2)	< .001

**Table 5:** Patient and operative characteristics in relation to mesh fixation, including unadjusted tests for significant differences

	Non-fixation		Tacks		Glue		*p*
	*n*	%	*n*	%	*n*	%	
ASA score							
I	3043	34.58	1864	29.18	1946	40.39	< .001
II	4737	53.84	3621	56.69	2461	51.08	
III/IV	1019	11.58	902	14.12	411	8.53	
Defect size							
I (< 1.5 cm)	1533	17.42	727	11.38	683	14.18	< .001
II (1.5–3 cm)	6072	69.01	3939	61.67	3200	66.42	
III (> 3 cm)	1194	13.57	1721	26.95	935	19.41	
EHS classification							
Combined	1128	12.82	633	9.91	394	8.18	< .001
Lateral	5483	62.31	3718	58.21	3142	65.21	
Medial	2188	24.87	2036	31.88	1282	26.61	
Drainage							
Yes	736	8.36	346	5.42	148	3.07	< .001
No	8063	91.64	6041	94.58	4670	96.93	
Risk factors^a^							
Total							
Yes	2248	25.55	1665	26.07	1233	25.59	0.747
No	6551	74.45	4722	73.93	3585	74.41	

^a^COPD, diabetes, aortic aneurysm, immunosuppression, corticoid medication, smoking, coagulopathy, antiplatelet medication, anticoagulation therapy

**Table 6:** Outcome variables in relation to mesh fixation, including unadjusted tests for significant differences

	Non-fixation		Tacks		Glue		p
	*n*	%	*n*	%	*n*	%	
Postoperative complications							
Total							
Yes	159	1.81	192	3.01	231	4.79	< .001
No	8640	98.19	6195	96.99	4587	95.21	
Bleeding							
Yes	76	0.86	50	0.78	34	0.71	0.602
No	8723	99.14	6337	99.22	4784	99.29	
Seroma							
Yes	61	0.69	133	2.08	189	3.92	< .001
No	8738	99.31	6254	97.92	4629	96.08	
Infection							
Yes	6	0.07	7	0.11	2	0.04	0.407
No	8793	99.93	6380	99.89	4816	99.96	
Bowel							
Yes	9	0.10	1	0.02	1	0.02	0.041
No	8790	99.90	6386	99.98	4817	99.98	
Wound healing disorders							
Yes	7	0.08	5	0.08	8	0.17	0.250
No	8792	99.92	6382	99.92	4810	99.83	
Ileus							
Yes	8	0.09	2	0.03	3	0.06	0.362
No	8791	99.91	6385	99.97	4815	99.94	

### Multivariable analysis

The results of multivariable analysis of the postoperative complications associated with seroma are summarized in Table 7 (model fit: *p* < 0.001). The fixation technique and hernia defect size exerted a highly significant influence on seroma formation (in each case *p* < 0.001). Glue compared to tacks (OR 2.077 [1.650; 2.613]; *p* < 0.001) and glue vs non-fixation (OR 5.448 [4.056; 7.317]; *p* < 0.001) resulted in an increased seroma rate. Similarly, tacks compared to non-fixation (OR 2.623 [1.925; 3.575]; *p* < 0.001) were associated with a higher risk of seroma formation. A large hernia defect (III vs. I: OR 2.868 [1.815; 4.531]; *p* < 0.001; II vs. I: OR 2.157 [1.410; 3.300]; *p* < 0.001) presented a significantly higher risk of seroma formation. That was also true for medial compared to lateral inguinal hernia (medial vs. lateral: OR 1.272 [1.020; 1.585]; *p* = 0.032), and for medial compared to combined (medial vs. combined: OR 2.194 [1.388; 3.470]; *p* < 0.001). Besides, a high ASA score (ASA III/IV vs. I: OR 1.645 [1.109; 2.442]; *p* = 0.013) led to an increased risk of seroma formation. The presence of risk factors (yes vs. no: OR 0.703 [0.543; 0.910]; *p* = 0.008) reduced the risk of seroma development. Only a trend towards a reduction in the risk of seroma formation was identified for the use of a drain (OR 0.584 [0.319; 1.072]; *p* = 0.083).

**Table 7:** Multivariable analysis of seroma formation following TAPP inguinal hernia repair

Parameter	*p* Value	Category	*p* Value paired	OR estimate	95%-CI	
Fixation	< .001	Glue vs. tacks	< .001	2.077	1.650	2.613
		Glue vs. non-fixation	< .001	5.448	4.056	7.317
		Tacks vs. non-fixation	< .001	2.623	1.925	3.575
Defect size	< .001	III (> 3 cm) vs. II (1.5–3 cm)	0.018	1.330	1.050	1.684
		III (> 3 cm) vs. I (< 1.5 cm)	< .001	2.868	1.815	4.531
		II (1.5–3 cm) vs. I (< 1.5 cm)	< .001	2.157	1.410	3.300
EHS classification	0.002	Lateral vs. combined	0.016	1.725	1.108	2.686
		Medial vs. lateral	0.032	1.272	1.020	1.585
		Medial vs. combined	< .001	2.194	1.388	3.470
Risk factors	0.008	Yes vs. no		0.703	0.543	0.910
ASA score	0.035	III/IV vs. II	0.166	1.258	0.909	1.742
		III/IV vs. I	0.013	1.645	1.109	2.442
		II vs. I	0.040	1.308	1.013	1.689
BMI (5-point OR)	0.069			0.861	0.733	1.011
Drainage	0.083	Yes vs. no		0.584	0.319	1.072
Age (10-year OR)	0.262			1.047	0.966	1.136
Mesh size (10-point OR)	0.597			0.985	0.930	1.043

## Discussion

This present analysis of data from the Herniamed Hernia Registry reveals highly significant differences in the postoperative complication rate in relation to the fixation technique for male patients with TAPP repair of primary unilateral inguinal hernia. For example, the overall postoperative complication rate for non-fixation was 1.8%, for tack fixation 3.0%, and for glue fixation 4.8% (*p* < 0.001). Seroma accounted for the greatest proportion of postoperative complications. Here, too, a highly significant difference was detected in relation to mesh fixation (non-fixation 0.7% vs. tacks 2.1% vs. glue 3.9%; *p* < 0.001). Multivariable analysis confirmed the highly significant influence exerted by the fixation technique on the seroma rate. Glue compared to tacks and non-fixation resulted in a higher seroma rate. But also tacks compared to non-fixation led to a higher seroma rate. Accordingly, no mesh fixation clearly had the lowest seroma rate. That could be one explanation for the lower seroma rates after TEP than after TAPP [8], since as per the guidelines [1, 2] mesh fixation is rarely used for TEP, whereas as evidenced by the present data this continues to be used in 65% of cases for TAPP [24]. But the evidence-based data demonstrate that even for TAPP mesh fixation can be omitted for hernia defects up to 3 cm (EHS LI, II and MI, II) [1, 2]. Based on the registry data only a defect size of more than 3 cm (EHS LIII, MIII), and here in particular medial and combined defects, requires mesh fixation [24]. Accordingly, as for TEP, mesh fixation can be dispensed with more often for TAPP, too. With a current proportion that continues to be as high as 65% [24], the fixation rate can still be markedly reduced for TAPP. Omission of fixation might then positively impact the seroma rate after TAPP.

Naturally, smaller defects also present a lower risk of seroma formation, as do lateral compared to medial inguinal hernias. Lateral defects in line with the anatomy of the groin have a curtain-like closure after excision of the hernia sac from the inguinal canal, whereas a medial hernia defect will persist as evagination of the transversalis fascia once repaired. It should therefore be reduced to prevent it from being filled with serous fluid. Various techniques have been proposed in the literature to that effect. Reddy [4] recommends the inversion of the extended transversalis fascia and fixation with tacks to the pubic bone. Technically, this is only feasible through the use of permanent tacks. Besides, the use of tacks can trigger chronic pain. Berney [5] recommends using a Röder loop, where the inverted transversalis fascia is ligated with the Röder loop such that the entrance to the medial hernia sac is closed and the sac completely reduced. Alternatively, the inverted transversalis fascia can be fixed to Cooper’s ligament with a suture and the hernia sac completely reduced in the same manner [25]. Using these techniques the problem of seroma formation can be completely prevented for a large medial inguinal hernia in both TEP and TAPP. Therefore this technique is urgently recommended in the guidelines [1, 2].

The aforementioned findings also highlight the potential role of drain placement for prevention of seromas. To date, only very few publications recommend the use of a drain [19, 20]. The results of multivariable analysis demonstrate a trend towards a protective role for a drain in reducing seroma formation after TAPP. But as arguments against drains complications and pain induced by drains must be mentioned. Since drains are used more commonly for TEP [25], this might also explain the difference in the seroma rate for TAPP vs. TEP.

There are reports in the literature that could explain the higher seroma rates identified on using glues. An experimental study with the cyanoacrylate-based tissue adhesives Glubran and Ifabond demonstrated that synthetic glues triggered evident seroma formation in the form of an inflammatory reaction [26]. This was caused by the toxic property of cyanoacrylate which, as far as possible, is reduced by means of chemical changes [26]. Some studies have reported that the fibrin glue Tisseel/Tissucol is associated with higher rates of seroma formation than staple fixation although that finding has been inconsistent [27]. It is likely that the avoidance of drop formation and local accumulation by spraying the fibrin sealant reduces the incidence of seroma formation [27].

The present study has a number of limitations. The registry does not contain any data on how the peritoneum was closed. Nor is any information given on whether the diagnosis “seroma” was based on physical examination or radiography. The possibility of confusing seromas with early hernia recurrences cannot be ruled out either.

In summary, it has been demonstrated that the seroma rate in male patients with TAPP repair of primary unilateral inguinal hernia is negatively influenced by mesh fixation with tacks or glue. Non-mesh fixation was associated with the lowest seroma rate. This can also explain the difference in the seroma rate between TEP and TAPP to the disadvantage of the latter technique since for TAPP mesh fixation continues to be used in 65% of cases. But the evidence-based data demonstrate that, as for TEP, mesh fixation should generally only be used, in TAPP too, for hernia defects of more than 3 cm (EHS LIII, MIII), and in particular for medial hernias. Otherwise, seroma formation is commonly encountered for large inguinal hernias, and especially for large medial inguinal hernias. Therefore medial defects should either be secured with a suture to Cooper’s ligament or reduced with a Roeder loop. Furthermore, there appears to be a trend towards a positive role for drain placement after TAPP in reducing seroma formation.

## Herniamed study group

### Scientific board

Köckerling, Ferdinand (Chairman) (Berlin); Bittner, Reinhard (Rottenburg); Fortelny, René (Wien); Jacob, Dietmar (Berlin); Koch, Andreas (Cottbus); Kraft, Barbara (Stuttgart); Kuthe, Andreas (Hannover); Lammers, Bernhard (Neuss); Lippert, Hans (Magdeburg); Lorenz, Ralph (Berlin); Mayer, Franz (Salzburg); Niebuhr, Henning (Hamburg); Peiper, Christian (Hamm); Pross, Matthias (Berlin); Reinpold, Wolfgang (Hamburg); Simon, Thomas (Weinheim); Stechemesser, Bernd (Köln); Unger, Solveig (Chemnitz); Weyhe, Dirk (Oldenburg); Zarras, Konstantinos (Düsseldorf).

### Participants

Ahmetov, Azat (Saint-Petersburg); Alapatt, Terence Francis (Frankfurt/Main); Albayrak, Nurretin (Herne); Amann, Stefan (Neuendettelsau); Anders, Stefan (Berlin); Anderson, Jürina (Würzburg); Antoine, Dirk (Leverkusen); Apfelstedt, Heinrich (Solingen); Arndt, Anatoli (Elmshorn); Aschenbrenner, Michael (Spittal/Drau); Asperger, Walter (Halle); Avram, Iulian (Saarbrücken); Baikoglu-Endres, Corc (Weißenburg i. Bay.); Bandowsky, Boris (Damme); Barkus; Jörg (Velbert); Becker, Matthias (Freital); Behrend, Matthias (Deggendorf); Berkhoff, Christian (Fulda); Beuleke, Andrea (Burgwedel); Birk, Dieter (Bietigheim-Bissingen); Bittner, Reinhard (Rottenburg); Blaha, Pavel (Zwiesel); Blumberg, Claus (Lübeck); Böckmann, Ulrich (Papenburg); Böhle, Arnd Steffen (Bremen); Bolle, Ludger (Berlin); Borchert, Erika (Grevenbroich); Born, Henry (Leipzig); Brabender, Jan (Köln); Breitenbuch von, Philipp (Radebeul); Brož, Miroslav (Ebersbach); Brückner, Torsten (Gießen); Brütting, Alfred (Erlangen); Buchert, Annette (Mallersdorf-Pfaffenberg); Buchholz, Torsten (Aurich); Budzier, Eckhard (Meldorf); Burchett, Bert (Teterow); Burghardt, Jens (Rüdersdorf); Cejnar, Stephan-Alexander (München); Chirikov, Ruslan (Dorsten); Claußnitzer, Christian (Ulm); Comman, Andreas (Bogen); Crescenti, Fabio (Verden/Aller); Daniels, Thies (Hamburg); Dapunt, Emanuela (Bruneck); Decker, Georg (Berlin); Demmel, Michael (Arnsberg); Descloux, Alexandre (Baden); Deusch, Klaus-Peter (Wiesbaden); Dick, Marcus (Neumünster); Dieterich, Klaus (Ditzingen); Dietz, Harald (Landshut); Dittmann, Michael (Northeim); Drummer, Bernhard (Forchheim); Eckermann, Oliver (Luckenwalde); Eckhoff (Jörn /Hamburg); Ehmann, Frank (Grünstadt); Eisenkrein, Alexander (Düren); Elger, Karlheinz (Germersheim); Engelhardt, Thomas (Erfurt); Erichsen, Axel (Friedrichshafen); Eucker, Dietmar (Bruderholz); Fackeldey, Volker (Kitzingen); Faddah, Yousif (Kamenz); Farke, Stefan (Delmenhorst); Faust, Hendrik (Emden); Federmann, Georg (Seehausen); Fiedler, Michael (Eisenberg); Fikatas, Panagiotis (Berlin); Firl, Michaela (Perleberg); Fischer, Ines (Wiener Neustadt); Fleischer, Sabine (Dinslaken); Fortelny, René H. (Wien); Franczak, Andreas (Wien); Franke, Claus (Düsseldorf); Frankenberg von, Moritz (Salem); Frehner, Wolfgang (Ottobeuren); Friedhoff, Klaus (Andernach); Friedrich, Jürgen (Essen); Frings, Wolfram (Bonn); Fritsche, Ralf (Darmstadt); Frommhold, Klaus (Coesfeld); Frunder, Albrecht (Tübingen); Fuhrer, Günther (Reutlingen); Garlipp, Ulrich (Bitterfeld-Wolfen); Gassler, Harald (Villach); Gawad, Karim A. Frankfurt/Main); Gehrig, Tobias (Sinsheim); Gerdes, Martin (Ostercappeln); Germanov, German (Halberstadt); Gilg, Kai-Uwe (Hartmannsdorf); Glaubitz, Martin (Neumünster); Glauner-Goldschmidt, Kerstin (Werne); Glutig, Holger (Meissen); Gmeiner, Dietmar (Bad Dürrnberg); Göring, Herbert (München); Grebe, Werner (Rheda-Wiedenbrück); Grothe, Dirk (Melle); Günther, Thomas (Dresden); Gürtler, Thomas (Zürich); Hache, Helmer (Löbau); Hämmerle, Alexander (Bad Pyrmont); Haffner, Eugen (Hamm); Hain, Hans-Jürgen (Gross-Umstadt); Halter, Christian Jörn (Recklinghausen); Hammans, Sebastian (Lingen); Hampe, Carsten (Garbsen); Hanke, Stefan (Halle); Harrer, Petra (Starnberg); Hartung, Peter (Werne); Heinzmann, Bernd (Magdeburg); Heise, Joachim Wilfried (Stolberg); Heitland, Tim (München); Helbling, Christian (Uznach/Schweiz); Hellinger, Achim (Fulda); Hempen, Hans-Günther (Cloppenburg); Henneking, Klaus-Wilhelm (Bayreuth); Hennes, Norbert (Duisburg); Herdter, Christian (Gelsenkirchen); Hermes, Wolfgang (Weyhe); Herzing, Holger (Höchstadt); Hessler, Christian (Bingen); Heuer, Matthias (Herten); Hildebrand, Christiaan (Langenfeld); Höferlin, Andreas (Mainz); Hoffmann, Henry (Basel); Hoffmann, Michael (Kassel); Hofmann, Eva M. (Frankfurt/Main); Horbach, Thomas (Fürth); Hornung, Frederic (Wolfratshausen); Hudak, Attila (Suhl); Hübel-Abe, Jan (Ilmenau); Hügel, Omar (Hannover); Hüttemann, Martin (Oberhausen); Hüttenhain, Thomas (Mosbach); Hunkeler, Rolf (Zürich); Imdahl, Andreas (Heidenheim); Iseke, Udo (Duderstadt); Isemer, Friedrich-Eckart (Wiesbaden); Jablonski, Herbert Gustav (Sögel); Jacob, Dietmar (Berlin); Jansen-Winkeln, Boris (Leipzig); Jantschulev, Methodi (Waren); Jenert, Burghard (Lichtenstein); Jugenheimer, Michael (Herrenberg); Junge, Karsten (Aachen); Kaaden, Stephan (Neustadt am Rübenberge); Käs, Stephan (Weiden); Kahraman, Orhan (Hamburg); Kaiser, Christian (Westerstede); Kaiser, Gernot Maximilian (Kamp-Lintfort); Kaiser, Stefan (Kleinmachnow); Karch, Matthias (Eichstätt); Kasparek, Michael S. (München); Kastl, Sigrid (Braunau am Inn); Keck, Heinrich (Wolfenbüttel); Keller, Hans W. (Bonn); Kewer, Jans Ludolf (Tuttlingen); Kienzle, Ulrich (Karlsruhe); Kipfmüller, Brigitte (Köthen); Kirsch, Ulrike (Oranienburg); Klammer, Frank (Ahlen); Klatt, Richard (Hagen); Klein, Karl-Hermann (Burbach); Kleist, Sven (Berlin); Klobusicky, Pavol (Bad Kissingen); Kneifel, Thomas (Datteln); Knolle, Winfried (Pritzwalk); Knoop, Michael (Frankfurt/Oder); Knotter, Bianca (Mannheim); Koch, Andreas (Cottbus); Koch, Andreas (Münster); Köckerling, Ferdinand (Berlin); Köhler, Gernot (Linz); König, Oliver (Buchholz); Kornblum, Hans (Tübingen); Krämer, Dirk (Bad Zwischenahn); Kraft, Barbara (Stuttgart); Kratsch, Barthel (Dierdorf/Selters); Krausbeck, Matthias (Schwerin); Kreissl, Peter (Ebersberg); Krones, Carsten Johannes (Aachen); Kronhardt, Heinrich (Neustadt am Rübenberge); Kruse, Christinan (Aschaffenburg); Kube, Rainer (Cottbus); Kühlberg, Thomas (Berlin); Kühn, Gert (Freiberg); Kuhn, Roger (Gifhorn); Kusch, Eduard (Gütersloh); Kuthe, Andreas (Hannover); Ladberg, Ralf (Bremen); Ladra, Jürgen (Düren); Lahr-Eigen, Rolf (Potsdam); Lainka, Martin (Wattenscheid); Lalla, Thomas (Oschersleben); Lammers, Bernhard J. (Neuss); Lancee, Steffen (Alsfeld); Lange, Claas (Berlin); Langer, Claus (Göttingen); Laps, Rainer (Ehringshausen); Larusson, Hannes Jon (Pinneberg); Lauschke, Holger (Duisburg); Lechner-Puschnig, Marina (Klagenfurt am Wörthersee/Österreich); Leher, Markus (Schärding); Leidl, Stefan (Waidhofen/Ybbs); Leisten, Edith (Köln); Lenz, Stefan (Berlin); Liedke, Marc Olaf (Heide); Lienert, Mark (Duisburg); Limberger, Andreas (Schrobenhausen); Limmer, Stefan (Würzburg); Locher, Martin (Kiel); Loghmanieh, Siawasch (Viersen); Lorenz, Ralph (Berlin); Luedtke, Clinton (Kusel); Luther, Stefan (Wipperfürth); Luyken, Walter (Sulzbach-Rosenberg); Mallmann, Bernhard (Krefeld); Manger, Regina (Schwabmünchen); Maurer, Stephan (Münster); May, Jens Peter (Schönebeck); Mayer, Franz (Salzburg); Mayer, Jens (Schwäbisch Gmünd); Mellert, Joachim (Höxter); Menzel, Ingo (Weimar); Meurer, Kirsten (Bochum); Meyer, Moritz (Ahaus); Mirow, Lutz (Zwickau); Mittag-Bonsch, Martina (Crailsheim); Möbius, Ekkehard (Braunschweig); Mörder-Köttgen, Anja (Freiburg); Moesta, Kurt Thomas (Hannover); Mugomba, Gilbert (Dannenberg); Moldenhauer, Ingolf (Braunschweig); Morkramer, Rolf (Radevormwald); Mosa, Tawfik (Merseburg); Müller, Hannes (Schlanders); Müller, Volker (Nürnberg); Münzberg, Gregor (Berlin); Murr, Alfons (Vilshofen); Mussack, Thomas (St. Gallen); Nartschik, Peter (Quedlinburg); Nasifoglu, Bernd (Ehingen); Neumann, Jürgen (Haan); Neumeuer, Kai (Paderborn); Niebuhr, Henning (Hamburg); Nix, Carsten (Walsrode); Nölling, Anke (Burbach); Nostitz, Friedrich Zoltán (Mühlhausen); Obermaier (Straubing); Öz-Schmidt, Meryem (Hanau); Oldorf, Peter (Usingen); Olivieri, Manuel (Pforzheim); Passon, Marius (Freudenberg); Pawelzik, Marek (Hamburg); Pein, Tobias (Hameln); Peiper, Christian (Hamm); Peiper, Matthias (Essen); Pertl, Alexander (Spittal/Drau); Philipp, Mark (Rostock); Pickart, Lutz (Bad Langensalza); Pizzera, Christian (Graz); Pöllath, Martin (Sulzbach-Rosenberg); Pöschmann, Enrico (Thalwil); Possin, Ulrich (Laatzen); Prenzel, Klaus (Bad Neuenahr-Ahrweiler); Pröve, Florian (Goslar); Pronnet, Thomas (Fürstenfeldbruck); Pross, Matthias (Berlin); Puff, Johannes (Dinkelsbühl); Rabl, Anton (Passau); Raggi, Matthias Claudius (Stuttgart); Rapp, Martin (Neunkirchen); Reck, Thomas (Püttlingen); Reinpold, Wolfgang (Hamburg); Renter, Marc Alexander (Moers); Reuter, Christoph (Quakenbrück); Radke, Alexander (Thun/Zweisimmen); Richter, Jörg (Winnenden); Riemann, Kerstin (Alzenau-Wasserlos); Riesener, Klaus-Peter (Marl); Rodehorst, Anette (Otterndorf); Roehr, Thomas (Rödental); Rössler, Michael (Rüdesheim am Rhein); Roncossek (Bremerhaven); Rosniatowski, Rolland (Marburg); Roth Hartmut (Nürnberg); Sardoschau, Nihad (Saarbrücken); Sauer, Gottfried (Rüsselsheim); Sauer, Jörg (Arnsberg); Seekamp, Axel (Freiburg); Seelig, Matthias (Bad Soden); Seidel, Hanka (Eschweiler); Seiler, Christoph Michael (Warendorf); Seltmann, Cornelia (Hachenburg); Senkal, Metin (Witten); Shamiyeh, Andreas (Linz); Shang, Edward (München); Siemssen, Björn (Berlin); Sievers, Dörte (Hamburg); Silbernik, Daniel (Bonn); Simon, Thomas (Weinheim); Sinn, Daniel (Olpe); Sinner, Guy (Merzig); Sinning, Frank (Nürnberg); Smaxwil, Constatin Aurel (Stuttgart); Sörensen, Björn (Lauf an der Pegnitz): Sucke, Jochen Markus (Gießen); Syga, Günter (Bayreuth); Schabel, Volker (Kirchheim/Teck); Schadd, Peter (Euskirchen); Schassen von, Christian (Hamburg); Schattenhofer, Thomas (Vilshofen); Scheibel, Mike (Krefeld); Schelp, Lothar (Wuppertal); Scherf, Alexander (Pforzheim); Scheuerlein, Hubert (Paderborn); Scheyer, Mathias (Bludenz); Schilling, André (Kamen); Schimmelpenning, Hendrik (Neustadt in Holstein); Schinkel, Svenja (Kempten); Schmid, Michael (Gera); Schmid, Thomas (Innsbruck); Schmidt, Ulf (Mechernich); Schmitz, Heiner (Jena); Schmitz, Ronald (Altenburg); Schöche, Jan (Borna); Schoenen, Detlef (Schwandorf); Schrittwieser, Rudolf (Bruck an der Mur); Schroll, Andreas (München); Schubert, Daniel (Saarbrücken); Schüder, Gerhard (Wertheim); Schürmann, Rainer (Steinfurt); Schultz, Christian (Bremen-Lesum); Schultz, Harald (Landstuhl); Schulze, Frank P. Mülheim an der Ruhr); Schulze, Thomas (Dessau-Roßlau); Schumacher, Franz-Josef (Oberhausen); Schwab, Robert (Koblenz); Schwandner, Thilo (Lich); Schwarz, Jochen Günter (Rottenburg); Schymatzek, Ulrich (Eitorf); Spangenberger, Wolfgang (Bergisch-Gladbach); Sperling, Peter (Montabaur); Staade, Katja (Düsseldorf); Staib, Ludger (Esslingen); Staikov, Plamen (Frankfurt am Main); Stamm, Ingrid (Heppenheim); Stark, Wolfgang (Roth); Stechemesser, Bernd (Köln); Steinhilper, Uz (München); Stengl, Wolfgang (Nürnberg); Stern, Oliver (Hamburg); Stöltzing, Oliver (Meißen); Stolte, Thomas (Mannheim); Stopinski, Jürgen (Schwalmstadt); Stratmann, Gerald (Goch); Straßburger, Harald (Alfeld); Stubbe, Hendrik (Güstrow/); Stülzebach, Carsten (Friedrichroda); Tepel, Jürgen (Osnabrück); Terzić, Alexander (Wildeshausen); Teske, Ulrich (Essen); Thasler, Wolfgang (München); Tichomirow, Alexej (Brühl); Tillenburg, Wolfgang (Marktheidenfeld); Timmermann, Wolfgang (Hagen); Tomov, Tsvetomir (Koblenz; Train, Stefan H. (Gronau); Trauzettel, Uwe (Plettenberg); Triechelt, Uwe (Langenhagen); Ulbricht, Wolfgang (Breitenbrunn); Ulcar, Heimo (Schwarzach im Pongau); Ungeheuer, Andreas (München); Unger, Solveig (Chemnitz); Utech, Markus (Gelsenkirchen); Verweel, Rainer (Hürth); Vogel, Ulrike (Berlin); Voigt, Rigo (Altenburg); Voit, Gerhard (Fürth); Volkers, Hans-Uwe (Norden); Volmer, Ulla (Berlin); Vossough, Alexander (Neuss); Wallasch, Andreas (Menden); Wallner, Axel (Lüdinghausen); Warscher, Manfred (Lienz); Warwas, Markus (Bonn); Weber, Jörg (Köln); Weber, Uwe (Eggenfelden); Weihrauch, Thomas (Ilmenau); Weiß, Heiko (Aue); Weiß, Johannes (Schwetzingen); Weißenbach, Peter (Neunkirchen); Werner, Uwe (Lübbecke-Rahden); Wessel, Ina (Duisburg); Weyhe, Dirk (Oldenburg); Wicht, Sebastian (Bützow); Wieber, Isabell (Köln); Wiens, Matthias (Affoltern); Wiesmann, Aloys (Rheine); Wiesner, Ingo (Halle); Withöft, Detlef (Neutraubling); Woehe, Fritz (Sangerhausen); Wolf, Claudio (Neuwied); Wolkersdörfer, Toralf (Pößneck); Yaksan, Arif (Wermelskirchen); Yildirim, Can (Lilienthal); Yildirim, Selcuk (Berlin); Zarras, Konstantinos (Düsseldorf); Zeller, Johannes (Waldshut-Tiengen); Zhorzel, Sven (Agatharied); Zuz, Gerhard (Leipzig);
